# The casein kinase MoYck1 regulates development, autophagy, and virulence in the rice blast fungus

**DOI:** 10.1080/21505594.2019.1649588

**Published:** 2019-08-08

**Authors:** Huan-Bin Shi, Nan Chen, Xue-Ming Zhu, Zhen-Zhu Su, Jiao-Yu Wang, Jian-Ping Lu, Xiao-Hong Liu, Fu-Cheng Lin

**Affiliations:** a State Key Laboratory of Rice Biology, Biotechnology Institute, Zhejiang University, Hangzhou, China; b State Key Laboratory of Rice Biology, China National Rice Research Institute, Hangzhou, China; c State Key Laboratory for Quality and Safety of Agro-products, Institute of Plant Protection Microbiology, Zhejiang Academy of Agricultural Science, Hangzhou, China; d College of Life Sciences, Zhejiang University, Hangzhou, China

**Keywords:** Magnaporthe oryzae, casein kinase, development, autophagy, virulence

## Abstract

Casein kinases are serine/threonine protein kinases that are evolutionarily conserved in yeast and humans and are involved in a range of important cellular processes. However, the biological functions of casein kinases in the fungus *Magnaporthe oryzae*, the causal agent of destructive rice blast disease, are not characterized. Here, two casein kinases, *MoYCK1* and *MoHRR25*, were identified and targeted for replacement, but only *MoYCK1* was further characterized due to the possible nonviability of the *MoHRR25* deletion mutant. Disruption of *MoYCK1* caused pleiotropic defects in growth, conidiation, conidial germination, and appressorium formation and penetration, therefore resulting in reduced virulence in rice seedlings and barley leaves. Notably, the *MoYCK1* deletion triggered quick lipidation of MoAtg8 and degradation of the autophagic marker protein GFP-MoAtg8 under nitrogen starvation conditions, in contrast to the wild type, indicating that autophagy activity was negatively regulated by MoYck1. Furthermore, we found that HOPS (homotypic fusion and vacuolar protein sorting) subunit MoVps41, a putative substrate of MoYck1, was co-located with MoAtg8 and positively required for the degradation of MoAtg8-PE and GFP-MoAtg8. In addition, *MoYCK1* is also involved in the response to ionic hyperosmotic and heavy metal cation stresses. Taken together, our results revealed crucial roles of the casein kinase MoYck1 in regulating development, autophagy and virulence in *M. oryzae*.

## Introduction

Rice blast disease is caused by the filamentous ascomycete fungus *Magnaporthe oryzae* (anamorph *Pyricularia oryzae*), posing serious threats to rice production and global food security [1,2]. Among a worldwide survey of the top ten fungal plant pathogens, *M. oryzae* was regarded as the most destructive fungal pathogen due to its economic and scientific significance [3]. The infection of *M. oryzae* starts upon three-cell conidia attaching to the host and germinating under proper conditions. Tips of the germ tube differentiate to form specialized and dome-shaped infection structures called appressoria, which are later melanized and accumulate substantial internal turgor to mechanically rupture the host cell wall [4]. Revealing the underlying developmental and infection mechanism of rice blast fungus could provide the potential to control rice blast disease more efficiently.

Casein kinase, first identified in rat livers, is named for its ability to phosphorylate casein [5]. According to conventional classification, casein kinases are divided into CKI groups related to the recognition of serine and threonine residues of substrates [6]. Members in this kinase family are rather few in contrast to other kinase groups. In wheat pathogenic fungus *Fusarium graminearum*, there are only two members [7]. Due to a whole-genome duplication, four casein kinases have evolved in *Saccharomyces cerevisiae* [8–10]. Current research in fungi has shown that casein kinases participate in a variety of cellular processes, including circadian rhythm regulation [11], intracellular vesicle transport [12], transcription [13], growth regulation [14], conidiation, morphogenesis [14], and virulence [7,15]. Evidence also suggests that casein kinases have functions in regulating several signaling pathways, including glucose-related sensing and signaling as well as Mpk1 and Hog1 MAPK signaling pathways [15,16].

Previous studies have demonstrated that autophagy is a key factor for the peak virulence of many plant fungal pathogens, including *M. oryzae* [17,18]. Autophagy is a conserved cellular process that is used by eukaryotes to engulf cargoes into double-membrane vesicles called autophagosomes, which ultimately fuse with vacuoles for degradation and recycling [19]. Upon autophagy induction, Atg8 protein is conjugated to the lipid phosphatidylethanolamine (PE) anchored in the autophagic membrane and is widely regarded as a marker of autophagy [20]. Autophagy is tightly regulated by two mechanisms, activation and inhibition. Excessive autophagy also leads to adverse pathological development, such as cell degeneration [21]. The negative regulation of autophagy has been intensively studied recently. In yeast, under nutrient availability, the TOR (target of rapamycin) kinase suppresses autophagy by phosphorylating Atg13 so it cannot interact with Atg1 to initiate autophagy [22]. In *M. oryzae*, MoSnt2, an epigenetic factor regulated by TOR, represses autophagy by modulating the deacetylation of histone 3 and the expression of the core autophagy-related genes *MoATG6, 15, 16* and *22* [23]. In addition, autophagy activity is controlled by posttranslational modification of autophagy core proteins. A recent study by Zhang *et al*. reported that histone acetyltransferase *GCN5* could inhibit light- and nitrogen-starvation-induced autophagy by acetylating Atg7 in *M. oryzae* [24]. Thus, declaring the mechanism underlying the negative regulation of autophagy becomes more important.

Vacuoles are intracellular degradation sites that correspond to lysosomes in mammals and are the most acidic organelles in fungi. In addition to their role in intracellular degradation and turnover, vacuoles are also implicated in signaling, ion homeostasis and osmotic regulation [25–27]. In the rice blast fungus, vacuoles are well known for lipid droplet degradation and thereby generate substantial turgor pressure in the appressorium [28]. In response to physiological conditions, vacuoles change in size and number (vacuolar fusion and fission) [29]. Vacuolar fusion is a complex process orchestrated by multiple factors, including the Rab GTPase Ypt7, soluble *N*-ethylmaleimide-sensitive factor attachment protein receptor (SNARE), SNARE complex disassembly chaperones, regulatory lipids, and the HOPS (homotypic fusion and vacuolar protein sorting) complex including Vps11, Vps16, Vps18, Vps33, Vps39 and Vps41 [30,31]. Functional analysis of several proteins involved in vacuolar membrane fusion in the plant pathogenic fungi *M. oryzae* and *F. graminearum*, including MoYpt7 [32], MoVam7 [33], MoMon1 [34], MoVps41 [35], FgYpt7 [36], FgVps41 [37], and FgMon1 [38], has established a link between vacuoles and fungal development and virulence. Functions of Vps41 homologs have been extensively explored in fungi, animals and plants and are implicated in vacuolar morphology, starvation response, intracellular survival, virulence, the endocytic pathway, antigen presentation and microbial killing [38–41]. In yeast, Vps41 is a substrate of the vacuolar casein kinase Yck3 and is an effector protein of the Rab GTPase Ypt7. The vacuolar casein kinase Yck3 and Ypt7 can orchestrate the localization of Vps41 to membrane fusion junctions [42]. The Ypt7 ortholog in *M. oryzae* and the Vps41 ortholog in *Aspergillus nidulans* have been indicated in the autophagy process as regulating membrane fusion between autophagosomes and vacuoles [32,43]. Therefore, the roles of Yck3 and Vps41 orthologs in *M. oryzae* in autophagy are worth investigating.

In the present study, we investigated the biological functions of *MoYCK1*, a putative casein kinase, in the rice blast fungus *M. oryzae* using a targeted knockout strategy. The *MoYCK1* deletion mutant showed defects in growth, asexual and sexual development, autophagy, hyperosmotic response, ion homeostasis, and virulence. We aim to provide some mechanistic insights into casein kinases in the filamentous fungal plant pathogen *M. oryzae*.

## Materials and methods

### Strains and growth conditions

The wild-type strain Guy11, its derivative null mutants Δ*Moyck1* and Δ*Movps41*, the complementation strains *Moyck1c* and *Movps41c*, and strains expressing GFP-*MoATG8* were used in this work. Strains were cultured on complete medium (CM), V8 medium, and OMA medium (OMA, 30 g oatmeal and 10 g agar in 1 L of distilled water) agar plates at 25°C with a 16-h light and 8-h dark cycle and stored on 5-mm filter paper disks at 20°C [44]. For mycelia collection, small agar blocks were taken from the edge of 7-day-old cultures before disrupting and culturing in liquid CM medium for 48 hours. To determine the utilization efficacy of carbon sources, strains were inoculated on minimal medium (MM) containing glucose or glucose-substituted MM with sucrose or mannose. To observe sexual reproduction, the wild-type strain Guy11 (Mat1-2), deletion mutant *ΔMoyck1* and complemented strain *Moyck1c* were inoculated crosswise with the opposite mating-type strain 2539 (Mat1-1) on oatmeal agar medium at 25°C for one week and then transferred to constant fluorescent light for another three weeks at 22°C [45].

### Stress response to heavy metal ions and osmotic reagents

To test the sensitivity of the mutants to metal ions, strains were inoculated on 1/4 YG medium (1.3 g/L yeast extract, 5 g/L glucose, 15 g/L agar) supplemented with 0.2 M CaCl_2_, 1 mM FeCl_2_, 0.5 mM CuCl_2_, 1 mM MnCl_2_ and 0.5 mM ZnCl_2_. To validate the growth of strains under hypertonic stress, strains Guy11, Δ*Moyck1* and *Moyck1c* were cultured on CM medium with 0.5 M NaCl, 0.7 M KCl, 1 M sorbitol and 1 M sucrose. Each strain was cultured with three independent replicates under each condition. The colony diameter was measured to calculate the relative inhibition rate at seven days post inoculation (dpi). In addition, 0.5 M NaCl was added to the liquid CM medium to detect the phosphorylation level of Osm1 at different time points under hypertonic conditions via western blotting with antibody (Cat. No. 9211S, Cell Signaling Technology, USA).

### Targeted gene deletion and complementation of deletion mutants

The construction of replacement vectors was performed according to previously described methods [46]. Two regions of approximately 1.0 kb of flanking sequences of the target genes were amplified with two pairs of specific primers (Table S1). The resulting PCR products were ligated with the hygromycin B phosphotransferase gene (*HPH*, used to delete *MoYCK1*) and the glufosinate acyltransferase gene (*BAR*, used to delete *MoVPS41)* and then inserted into *XbaI/HindIII* sites of the vector pKO1B using a one-step cloning kit (Cat. No. C113-02, Vazyme, China). Then, the wild-type Guy11 strain was transformed with the correct knockout vectors by inserting a marker gene cassette into the two flanking sequences of the target genes via *Agrobacterium tumefaciens*-mediated transformation (ATMT) [47]. Putative deletion transformants without green fluorescence were further verified by PCR and Southern blotting.

To generate complemented constructs of *pKD5-MoVPS41* or *pKD5-MoYCK1*, the alleles of wild-type *MoVPS41* or *MoYCK1* were amplified and fused to *EcoRI/BamHI*-digested pKD5 [48], which contains the sulfonylurea resistance gene. The resulting vector was transferred into *ΔMovps41* or *ΔMoyck1* by ATMT, and transformants were screened on DCM medium (10 g/L glucose, 2 g/L asparagine, 1.7 g/L yeast nitrogen base without amino acid, 1 g/L NH_4_NO_3_, and 15 g/L agar, pH 6.0 adjusted with NaH_2_PO_4_) with 100 µg/ml sulfonylurea.

### Southern blot analysis

The genomic DNA samples from Guy11 and the Δ*Moyck1 *mutant were extracted and digested with *BamHI*. The digest products were separated on a 0.7% agarose gel and then transferred to a nitrocellulose membrane. The *MoYCK1* gene probe was amplified from Guy11 genomic DNA using primers *MoYCK1*-probeF/*MoYCK1*-probeR. To confirm replacements of *MoYCK1*, a DIG-labeled *MoYCK1* probe was used to hybridize with the *BamHI*-digested genomic DNA from the Δ*Moyck1* mutant and wild-type Guy11. The whole hybridization was carried out according to the manufacturer’s instructions for the DIG-High Prime system (Cat. No. 11745832910, Roche, Germany) [49]. The same manipulation was adopted to demonstrate a replacement of the *MoVPS41* gene.

### Phenotypic analysis

The growth characteristics of *M. oryzae* strains were analyzed according to previously described methods [46]. Each experiment was repeated three times with either three or five replicates. The 5-mm mycelial blocks of Guy11 and derivative strains cultured for 7 days were inoculated onto 6-cm-diameter CM plates. The colony diameter and spore production were measured at seven dpi. The conidial size and morphology were surveyed on more than 200 conidia separately after staining the cell walls of spores with CFW (10 µg/mL). Conidiophore development was monitored according to a previous description [50]. For conidial germination and appressorium formation assays, 40 μL of spore suspension (1 × 10^5^ conidia/mL) was inoculated on a hydrophobic plastic coverslip and incubated in a moist chamber at 28°C in the dark. The conidial germination rate or appressorium formation rate of 100 conidia was evaluated at 4 and 24 hours post incubation (hpi).

### Pathogenicity assay

For plant infection assays, conidia harvested from Guy11, Δ*Moyck1* and *Moyck1c* were suspended in a 0.2% (w/v) gelatin solution (1 × 10^4^ conidia/mL), and then two milliliters of each solution was sprayed onto 3–4 leaf-stage seedlings of susceptible rice (*Oryza sativa* cv. CO39). The results were photographed after 5 days post inoculation under humid conditions at 25°C [51]. To analyze the pathogenicity of mutants on detached barley leaves, 20 μL droplets of the conidia suspensions (1 × 10^4^ conidia/mL) were placed onto the upper side of the 7-day-old barley leaves. The leaves were decolored and observed at 48 hpi to investigate appressorium-mediated penetration and invasive growth of Guy11 and derivative mutants [52].

### Western blot analysis

To collect mycelia, the wild-type strain Guy11 and the derivative mutant strains were cultured in liquid CM for 48 hours before collection. For autophagy induction by nitrogen starvation, mycelia were transferred into MM-N for 3 or 6 hours. Total proteins were suspended in protein extraction buffer after grinding [53]. After protein sample quantification with a BCA protein content assay kit (Cat. No. C503021, Beyotime, China), equal quantities of total proteins were loaded and separated on a 12% SDS-polyacrylamide gel and then transferred to a PVDF membrane. The membrane was blocked and incubated with primary anti-GFP (Cat. ab32146, abcam, UK) and secondary antibody (HRP-conjugated peroxidase) sequentially. The ECL chemiluminescent kit (Cat. No. 1,705,060, BioRad, USA) was used for western blotting detection. Lipidation of MoAtg8 was detected with antibody anti-Atg8 (Cat. No. PM090, MBL, Japan) according to a previously described method [54]. GAPDH was used as a loading control with the anti-GAPDH antibody (Cat. No. R1208-3, HuaBio, China).

### Fluorescence observation

To monitor autophagic affluxes, a GFP*-MoATG8* fusion protein driven by its native promoter was transferred into Guy11, Δ*Moyck1* and Δ*Movps41* via ATMT as previously described [32]. Transformants with green fluorescence were used for observation. The expression of GFP and *MoATG8* was confirmed with qPCR as previously described. Strains were treated with nitrogen starvation before observation under a confocal fluorescence microscope (Zeiss LSM780). To visualize the vacuole, 10 µm CMAC (7-amino-4-chloromethylcoumarin) was added to the conidial suspension and incubated for 30 min before imaging.

To visualize the colocalization of MoVps41 and MoAtg8, the coding sequence of the *MoVPS41* gene was amplified from the mycelial cDNA library of Guy11 with primers VPS41GFPF/VPS41GFPR and ligated into the *SmaI*-digested pKD5-GFP vector with a constitutive H3 promoter from *M. oryzae* histone 3 protein [35]. The resulting vector pKD5-MoVps41GFP was transformed into *ΔMovps41* by ATMT. Transformants were screened on DCM medium containing 100 µg/ml sulfonylurea. A previously constructed vector, pDsRed2-MoAtg8 [55], was introduced into transformants expressing MoVps41GFP. Fluorescence localization in the mycelial stage was observed by incubating the conidia in liquid CM for 24 hours. Fluorescent signals were observed with ZEN software using a Zeiss LSM780 inverted confocal laser scanning microscope equipped with a 30 mW argon laser and 63× oil objective.

## Results

### Identification and disruption of casein kinases Moyck1 and MoHRR25 in *M. oryzae*

**Figure 1.**
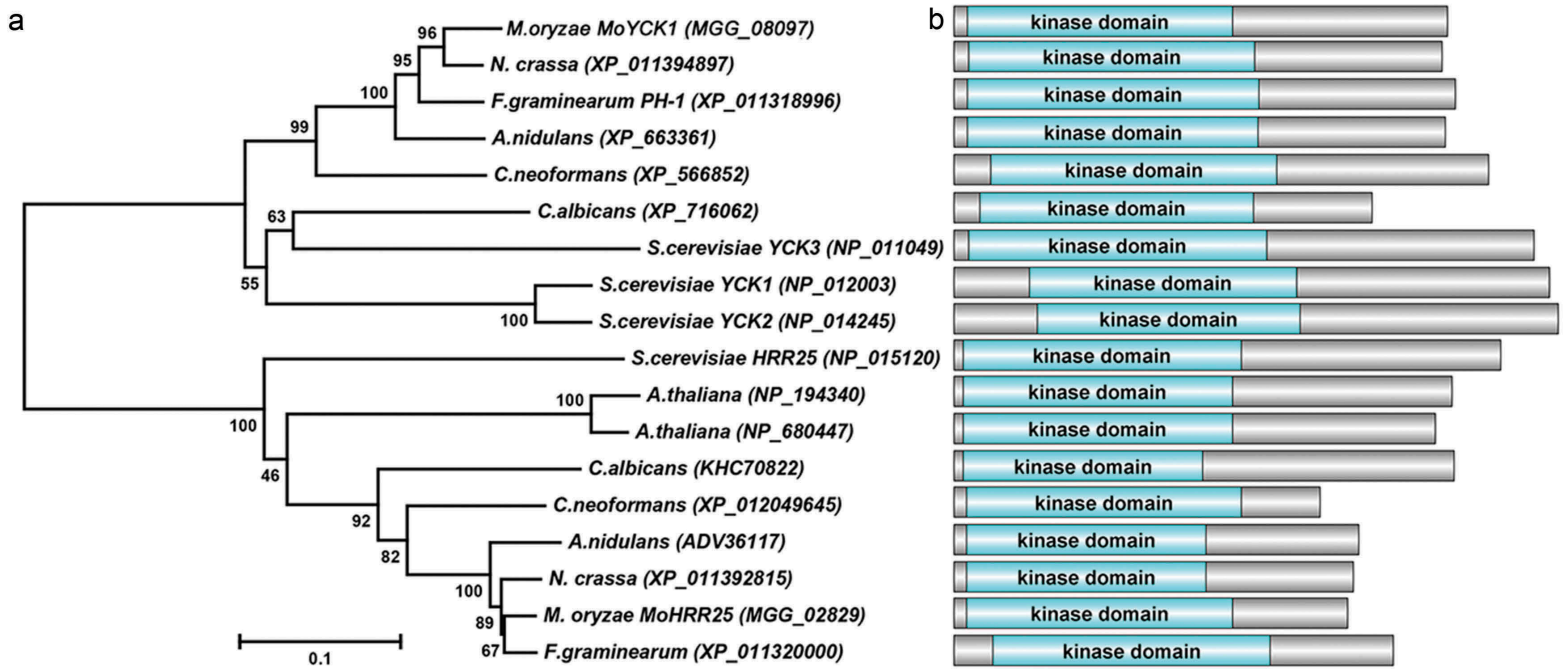
Phylogenetic tree analysis and domain prediction among casein kinase homologs. (a). The phylogenetic tree was constructed by MEGA 5.0 using the neighbor-joining tree construction method with 1000 bootstrap replicates. Sequences of casein kinase homologs were obtained from NCBI databases and aligned by Clustal Omega. Accession numbers of homologs are indicated in the figure. (b). Domains contained in casein kinases were predicted using the SMART webserver. The picture was drawn using DOG 2.0 software.

To identify orthologs of yeast casein kinases in *M. oryzae*, the protein sequences of *S. cerevisiae* casein kinases Yck1, Yck2, Yck3, and Hrr25 were used as the query sequences for protein BLAST comparisons with the NCBI database of *M. oryzae*. The search results for *S. cerevisiae* casein kinases Yck1, Yck2, and Yck3 identified the same gene (MGG_08097), which shows 70.3%, 71.3%, and 60.9% identity with ScYck1, ScYck2, and ScYck3, respectively. Thus, MGG_08097 was named *MoYCK1*. MGG_02829 shows 70.7% identity in the amino acid sequence with ScHrr25; therefore, we named it *MoHRR25*. A phylogenetic analysis of casein kinase homologs from fungi and plants revealed that *MoYCK1* and *MoHRR25* show a close evolutionary relationship to homologs in *N. crassa* and *F. graminearum*, respectively. While both *MoYCK1* and *MoHRR25* display distant relationships with yeast homologs, they still have high similarities. Functional domain prediction of casein kinase homologs revealed that they contain a conserved kinase domain located at the nitrogen terminus of the coding sequences (Figure 1).

**Figure 2.**
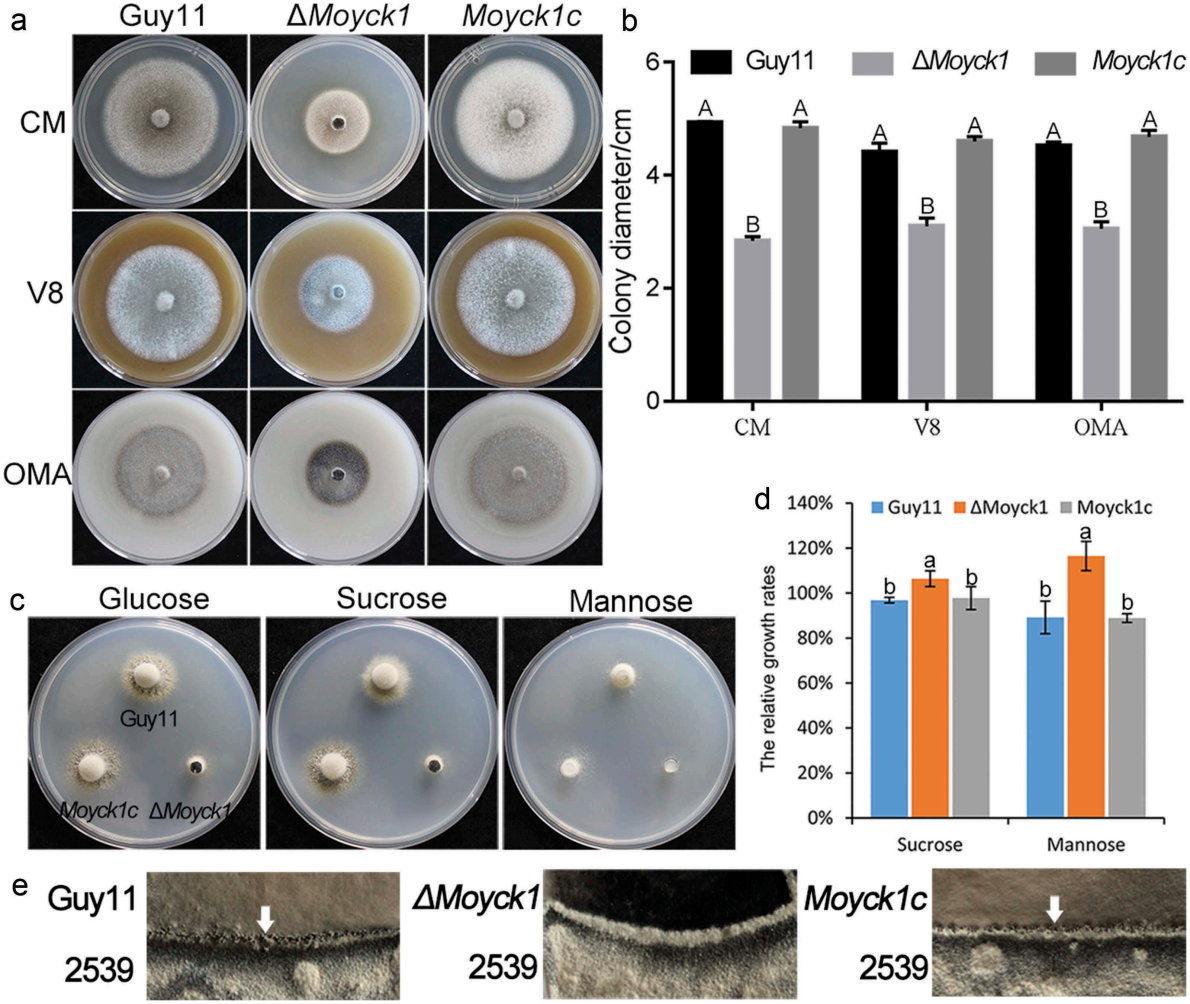
MoYck1 is involved in growth and sexual reproduction in *M. oryzae*. (a). Growth of Guy11, Δ*Moyck1* and *Moyck1c* on CM, V8, and OMA plates. Mycelial plugs were inoculated on the above plates for 8 days before photography. (b). Diameters of colonies were measured and mean and standard deviation were presented calculated from data of three replicates. The same characteristic showed no significant differences (Duncan’s test, P < 0.01), and the error bars represent the standard deviation. (c). Utilization of different carbon sources. Mycelial plugs of Guy11, Δ*Moyck1* and *Moyck1c* were inoculated on MM plates with glucose, sucrose, and mannose for 7 days before photography. (d). Relative growth ratios of Guy11, Δ*Moyck1* and *Moyck1c*. The averages are calculated from measuring three replicates, representing relative ratios of growth on sucrose and mannose relative to that on glucose. The data is subject to Duncan’s test and a significant difference is shown in the figure (P < 0.05). (e). Sexual development. Crossing of Guy11 or Δ*Moyck1* backcrossed with the opposite mating strain 2539 under constant fluorescent light at 20°C to induce sexual development. Arrows indicate the formation of perithecia at the junction of Guy11 and 2539.

To determine the biological roles of *MoYCK1* and *MoHRR25*, knockout vectors carrying the hygromycin resistance gene *HPH* were constructed and transferred into the wild-type strain, respectively. The *MoYCK1* deletion mutant Δ*Moyck1* was successfully obtained via PCR and Southern blotting verification (Fig. S1). However, we failed to obtain a *MoHRR25* null mutant after repeat knockout three times. This failure may be due to its essential role in viability. We gained the complemented strain *Moyck1c* by reintroduction of the wild-type *MoYCK1* allele into Δ*Moyck1*. In *Moyck1c*, defects in growth, conidiation and virulence were restored to comparable levels of the wild type (Figure 2(a)).

### Deletion of Moyck1 affects vegetative growth and sexual development

In contrast to the growth of the wild-type and complemented strains, Δ*Moyck1* grew slowly on complete medium, V8 medium, and OMA plates, respectively, at 7 days post inoculation. In yeast, ScYck1/2 is involved in glucose signaling and hexose transport [16]. To evaluate the sensing and utilization of different carbon sources, mycelial plugs of the wild-type strain Guy11, Δ*Moyck1* and the complemented strain *Moyck1c* were inoculated on MM and glucose-substituted MM with sucrose or mannose agar plates for 7 days. As shown in Figure 2(a and b), the colony diameter of the Δ*Moyck1 *mutant was clearly smaller than that of the wild-type and complemented strains on each kind of plate, but Δ*Moyck1* displayed higher efficacy in utilization of carbon sources than glucose, in contrast to the wild type and *Moyck1c* (Figure 2(c and d)). These data indicated that *MoYCK1* is responsible for hyphal growth and glucose utilization.

In *C. neoformans*, Cck1, the homolog of MoYck1, can regulate the mating process [15]. To determine the role of *MoYCK1* in *M. oryzae* sexual development, Guy11, Δ*Moyck1* and the complemented strain *Moyck1c* were crossed with the opposite mating strain 2539 on OMA plates for 4 weeks. Guy11 and complemented strain *Moyck1c* developed numerous perithecia, but no perithecia were formed at the junction between Δ*Moyck1* and 2539 (Figure 2(e)), suggesting that *MoYCK1* plays essential roles in sexual development.

### Moyck1 is required for conidiogenesis and conidial morphology

**Figure 3.**
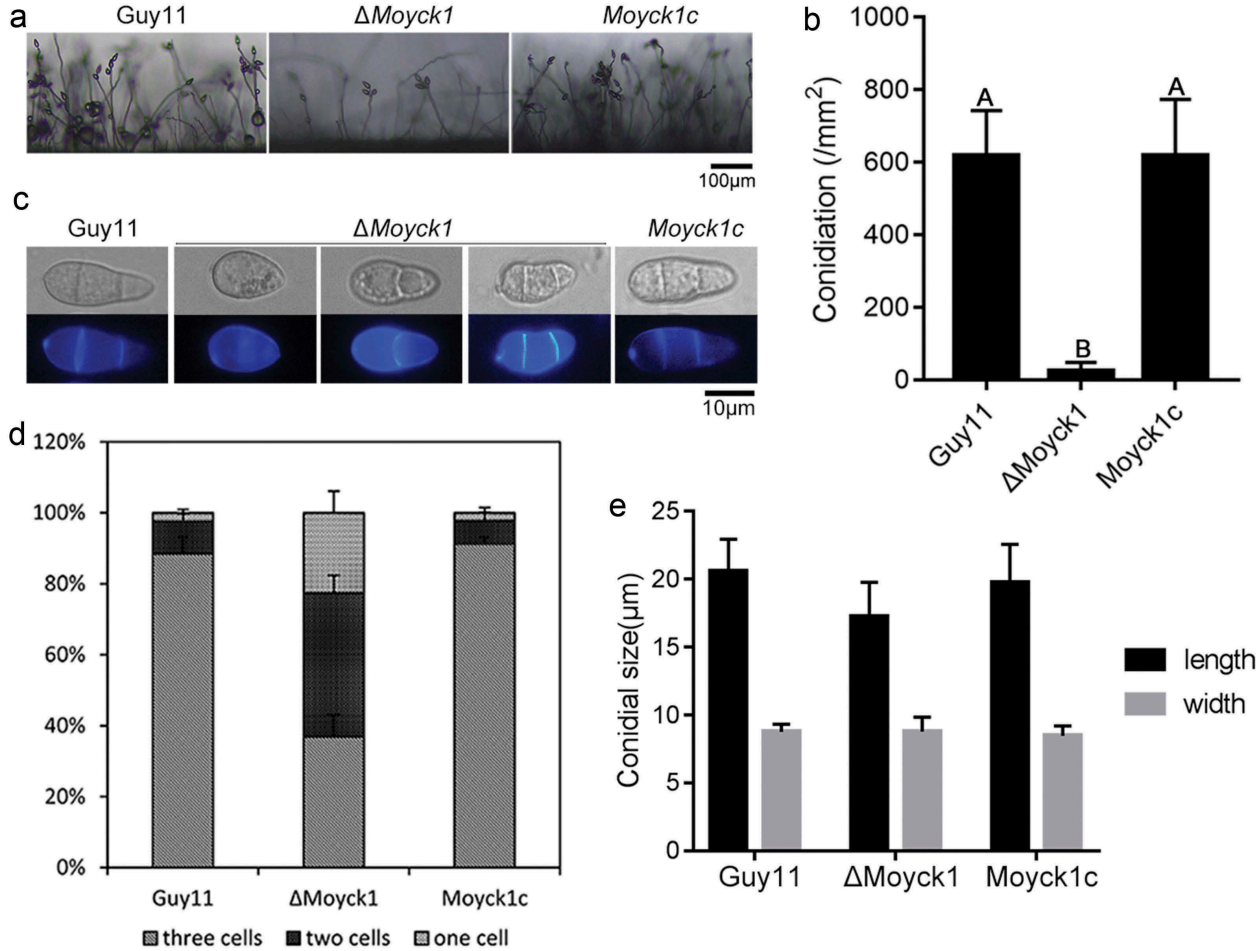
MoYck1 is required for conidiogenesis and conidial morphology. (a). Observation of conidiophore differentiation. Bar = 100 µm. (b). Conidiation was measured at 7 dpi on CM plates. C. CFW staining. Conidia from Guy11, Δ*Moyck1* and *Moyck1c* were stained by CFW. (d). Conidial septation. The proportions of conidia with various numbers of septa were counted. (e). Size of conidia. The length and width of more than two hundred conidia were measured. Different letters indicate a significant difference by Duncan’s test (P < 0.01), with error bars representing the standard deviation.

To evaluate the effects of *MoYCK1* on fungal conidiation, conidia from Guy11, Δ*Moyck1* and complemented strain *Moyck1c* were collected when cultured on CM plates at 7 days post incubation. The results showed that Δ*Moyck1* had severely reduced conidiation compared to the wild-type and complemented *Moyck1c* strains. (Figure 3(b)). Further microscopic observation of conidiophores also showed that Δ*Moyck1* produced few conidiophores after 24 hours of induction under continuous light exposure. In contrast, under the same induction conditions, numerous conidiophores were differentiated in both the wild-type strain and the complemented strain (Figure 3(a)).

Interestingly, we found via CFW staining that conidial morphology was significantly altered in the null mutant Δ*Moyck1*. There was a higher proportion of conidia with no septum or one septum in Δ*Moyck1* compared with those in Guy11 and the complemented strain *Moyck1c* (Figure 3(c and d)). In addition, conidia in Δ*Moyck*1 were shorter than those in Guy11 and *Moyck1c*, although they did not display a difference in conidial width (Figure 3(e)). Overall, our observations indicated that *MoYCK1* plays important roles in asexual development of *M. oryzae*.

### Moyck1 is involved in conidial germination and appressorium formation

**Figure 4.**
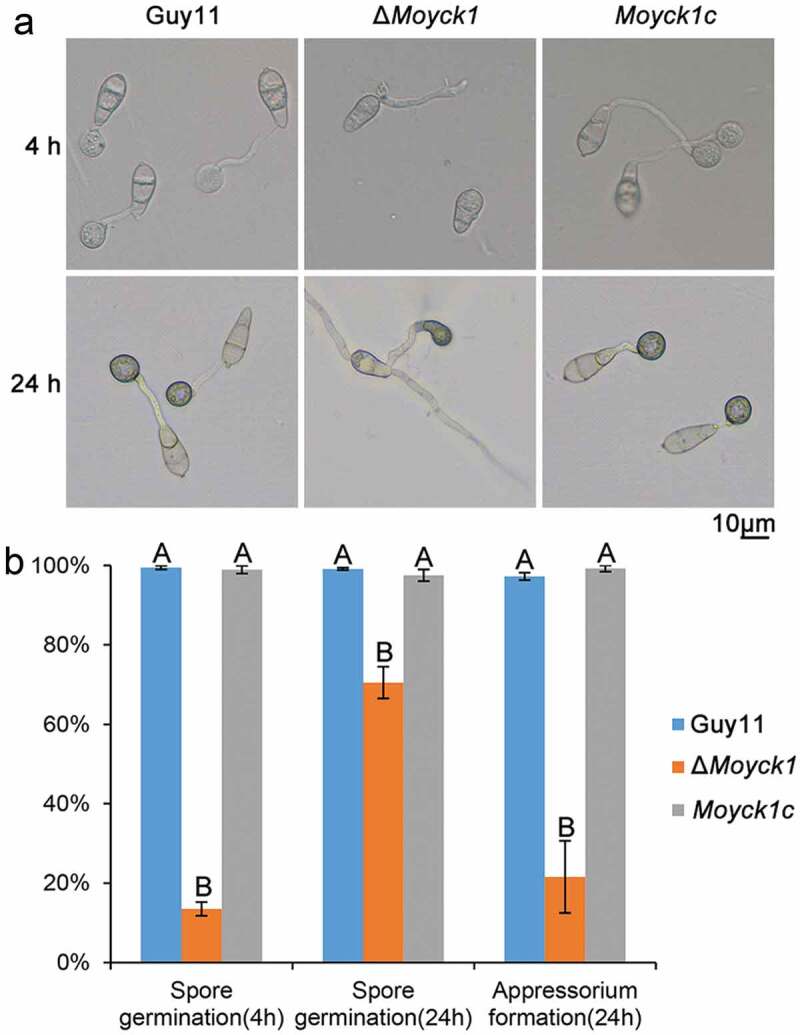
MoYck1 is responsible for conidial germination and appressorium formation. (a). Conidial germination was observed at 4 and 24 hours post incubation on the hydrophobic surface. (b). Ratios of germination and appressorium formation were counted. The data with the same characters indicate no significant differences (Duncan’s test, P < 0.01).

To determine whether MoYck1 also participates in the early infection development process, the ratios of conidial germination and appressorium formation in Guy11, Δ*Moyck1* and *Moyck1c* were measured at 4 or 24 hpi on hydrophobic coverslips. At 4 hpi, only 13.5 ± 1.7% of conidia from Δ*Moyck1* could germinate, in contrast to 99.5 ± 0.5% in Guy11 and 98.9 ± 1% in *Moyck1c*. The proportion of germination in Δ*Moyck1* increased to 70.5 ± 4% at 24 hpi, but there were apparent differences from those of Guy11 and *Moyck1c* (Figure 4). In addition, appressorium formation was severely impaired in Δ*Moyck1* (21.5 ± 9%) at 24 hpi, while conidia in Guy11 (97.2 ± 1%) and in the complemented strain *Moyck1c* (99.2 ± 1%) could form appressoria (Figure 4). These data demonstrated that MoYck1 is essential for conidial germination and appressorium formation in *M. oryzae.*

### Moyck1 contributes to full virulence

**Figure 5.**
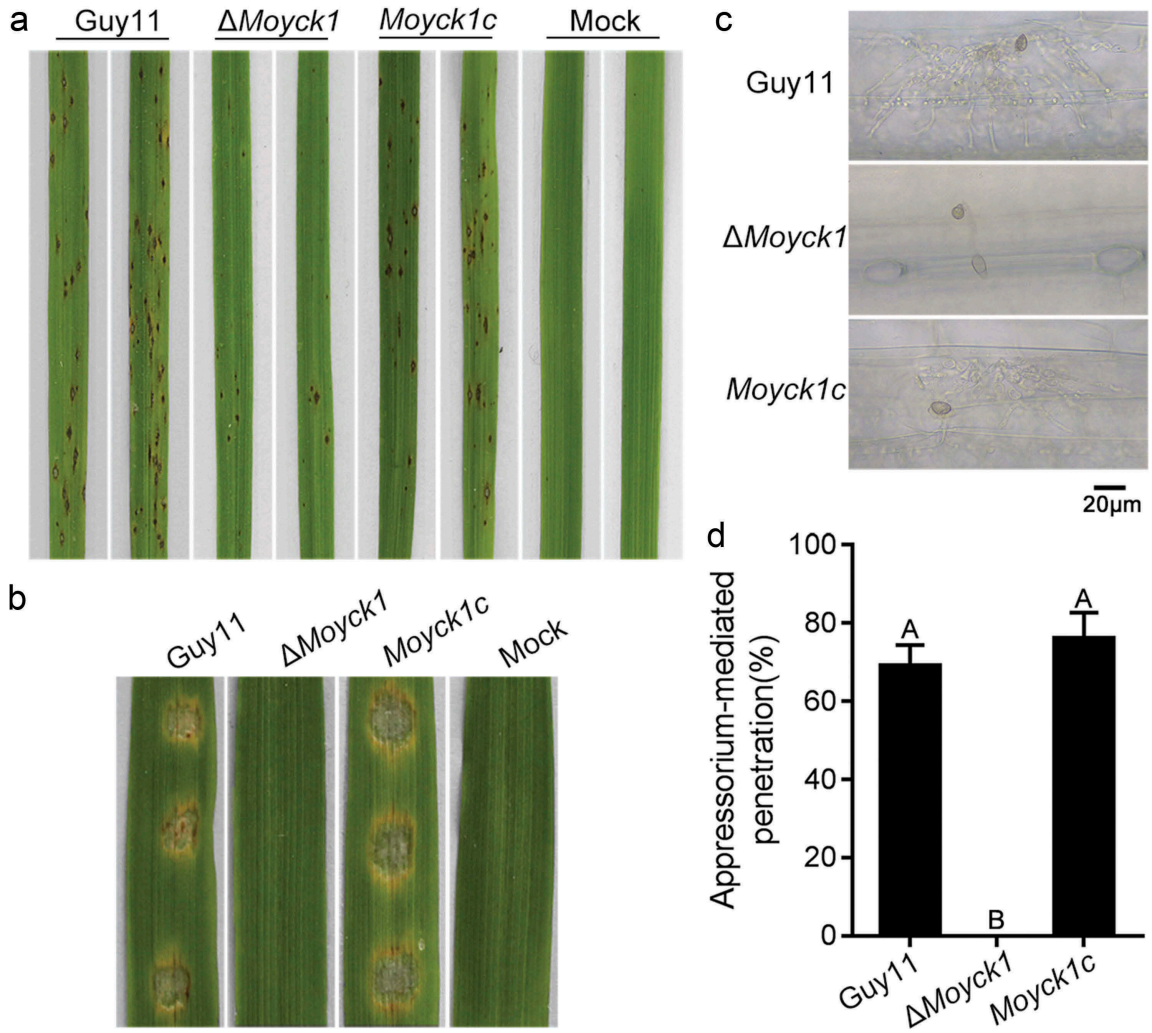
MoYck1 contributes to full virulence. (a). Virulence on rice seedlings. Conidia (1 × 10^4^/mL) from Guy11, Δ*Moyck1* and *Moyck1c* were sprayed on 2-week-old rice seedlings, and pictures were taken at 5 dpi. (b). Virulence on barley leaves. Conidial drops were inoculated on barley leaves *in vitro*. Photos were taken at 5 dpi. (c). Appressorium-mediated penetration on barley leaves. The barley leaves were inoculated with conidia for 48 hours and then decolored before observation. Bar = 20 µm. (d). Ratios of appressorium-mediated penetration. The values mean average and bars mean standard errors. The column labeled with different characters means an apparent difference (Duncan’s test, P < 0.01).

To determine the role of MoYck1 in virulence, two-week-old rice seedlings were inoculated with conidial suspensions from Guy11, Δ*Moyck1* and the complemented strain *Moyck1c*. The null mutant Δ*Moyck1* caused few lesions on seedling leaves than those of Guy11 and *Moyck1c* at 5 dpi (Figure 5(a)). Furthermore, equal amounts of conidia from Guy11, Δ*Moyck1* and the complemented strain *Moyck1c* were inoculated on barley leaves *in vitro*. Similar results were obtained (Figure 5(b)), suggesting that MoYck1 is essential for plant infection in *M. oryzae*. In addition, microscopic observation confirmed that appressoria formed in Δ*Moyck1* could not penetrate into plant cells such as those formed in Guy11 and the complemented strain *Moyck1c* (Figure 5(c and d)), suggesting a defect in appressorium maturation in Δ*Moyck1*.

### Moyck1 negatively regulates autophagy activity

**Figure 6.**
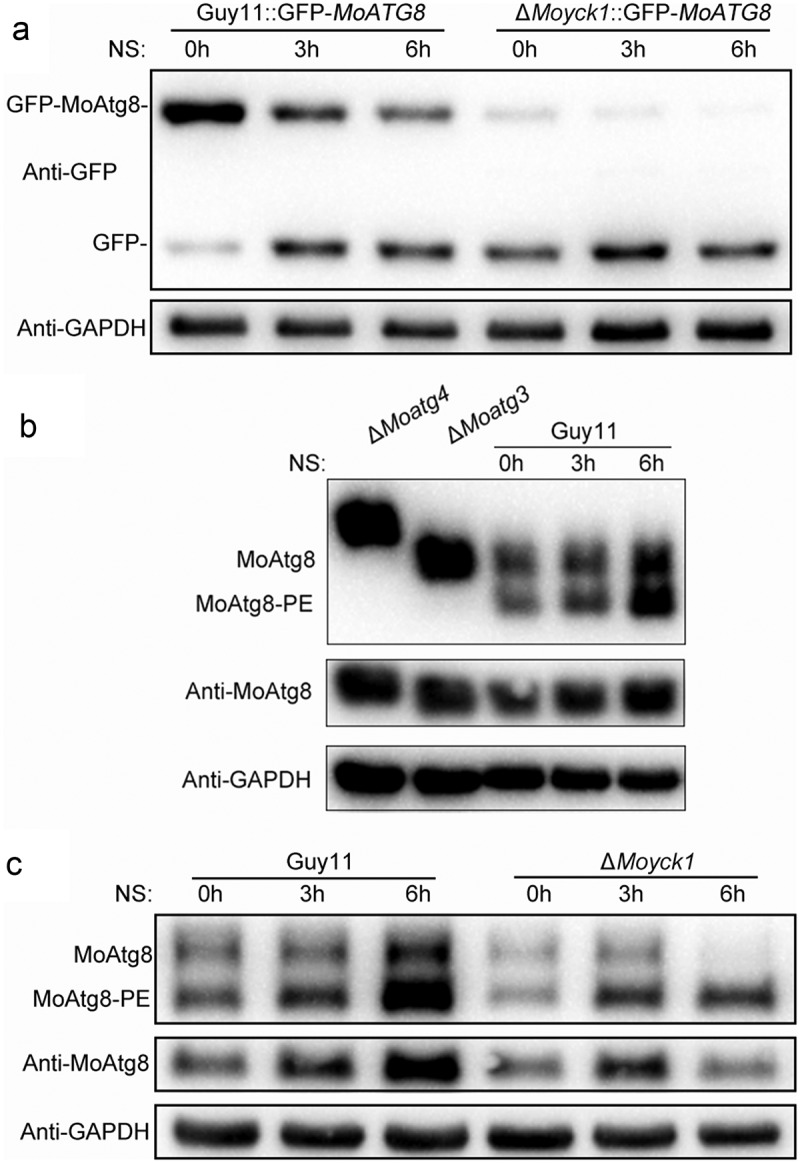
Autophagy is negatively regulated by MoYck1 in *M. oryzae*. (a). Observation of autophagic affluxes in Guy11 and Δ*Moyck1*. The degradation of GFP-MoAtg8 was observed via western blotting with an anti-GFP antibody. GAPDH was used to indicate the loading amount of total protein. (b). Lipidation of MoAtg8 observed in *M. oryzae*. Lipidation of MoAtg8 was observed in Δ*Moatg4*, Δ*Moatg3* and Guy11 under nitrogen starvation conditions for 3 and 6 hours via western blotting with anti-MoAtg8. (c). Lipidation of MoAtg8 compared with that in Guy11 and Δ*Moyck1*. Lipidation of MoAtg8 and the amount of MoAtg8 were observed in Guy11 and Δ*Moyck1*. The protein GAPDH was used as a loading control.

Autophagy is a vacuole-dependent degradation system. Vacuole morphology maintains tight relationships with autophagy activity [29]. In *S. cerevisiae*, Yck3 and the Rab GTPase Ypt7 control vacuole membrane fusion by regulating the downstream effector protein Vps41, a HOPS subunit involved in membrane tethering and fusion [56]. Recently, MoYpt7 and MoVps41, homologs of *S. cerevisiae* Ypt7 and Vps41, have been found to be essential for vacuole morphology [32,35]. To determine whether MoYck1 is involved in the autophagy process, autophagy levels in the wild-type strain and in Δ*Moyck1* were compared by detecting the degradation of GFP-MoAtg8, an autophagic marker protein, to measure autophagic affluxes. The results showed that the fusion protein GFP-MoAtg8 could be normally degraded in the Guy11 and Δ*Moyck1* backgrounds, but the degradation rate of GFP-MoAtg8 in Δ*Moyck1* was faster than that of Guy11 (Figure 6(a)), indicating that the autophagy process remained higher after deletion of *MoYCK1*. Consistent with this presumption, the initial protein amount of GFP-MoAtg8 was less in Δ*Moyck1* than in Guy11. To determine whether the difference in degradation is attributable to affected GFP-*MoATG8* transcription in Guy11 and Δ*Moyck1*, expression levels of GFP-*MoATG8* in both strains were detected via qRT-PCR. The results showed that GFP-*MoATG8* gene expression levels were comparable in Guy11 and Δ*Moyck1* when cultured in sufficient nutrient CM medium. However, upon transfer to nitrogen-starvation conditions, GFP*-MoATG8* expression levels in Δ*Moyck1* were higher (>2-fold) than in Guy11 (Fig. S2). These data indicated that MoYck1 negatively controls autophagy.

In *S. cerevisiae*, during autophagy induction, Atg8 is first cleaved by the cysteine protease Atg4 to expose its carboxyl glycine and then subjected to lipidation catalyzed by sequential enzymes, including ubiquitin-activating enzyme-like Atg7, ubiquitin-conjugating enzyme-like Atg3 and ubiquitin ligase-like Atg16-Atg5-Atg12 complex, and ultimately bound to membrane-anchored phosphatidylethanolamine (PE) [57]. To determine whether MoYck1 is involved in the early stage of autophagy, lipidation of MoAtg8 was investigated in Guy11, Δ*Moyck1* and *Moyck1c* via western blotting. First, we observed the lipidation process of MoAtg8 in Δ*Moatg4*, Δ*Moatg3*, and Guy11 treated with nitrogen starvation for 0, 3 and 6 hours. In Δ*Moatg4*, MoAtg8 could not be cleaved and lipidated, while cleaved MoAtg8 failed to be linked to PE in Δ*Moatg3*. In contrast, lipidized MoAtg8 could be generated in Guy11 and increased with prolonged nitrogen starvation treatment (Figure 6(b)). These data confirmed that the molecular mechanism underlying the lipidation of MoAtg8 is conserved in *M. oryzae*. Then, the lipidation process was compared in Guy11 and Δ*Moyck1* according to the above experimental system. With extended nitrogen starvation, both the protein level and lipidation level of MoAtg8 in Guy11 increased. However, the protein level of MoAtg8 in Δ*Moyck1* increased at 3 hpi but decreased at 6 hpi. In addition, MoAtg8 in Δ*Moyck1* was almost in a lipidized state at 6 hours postinduction (Figure 6(c)). We also detected the gene expression level of MoAtg8 in Guy11, Δ*Moyck1* and *Moyck1c*. The results suggested that similar expression patterns were present in Guy11, Δ*Moyck1* and *Moyck1c*, with an upregulation trend during nitrogen starvation (Fig. S3). These results indicated that MoYck1 plays a negative role in regulating MoAtg8 lipidation.

### Moyck1 might regulate autophagy by MoVps41

**Figure 7.**
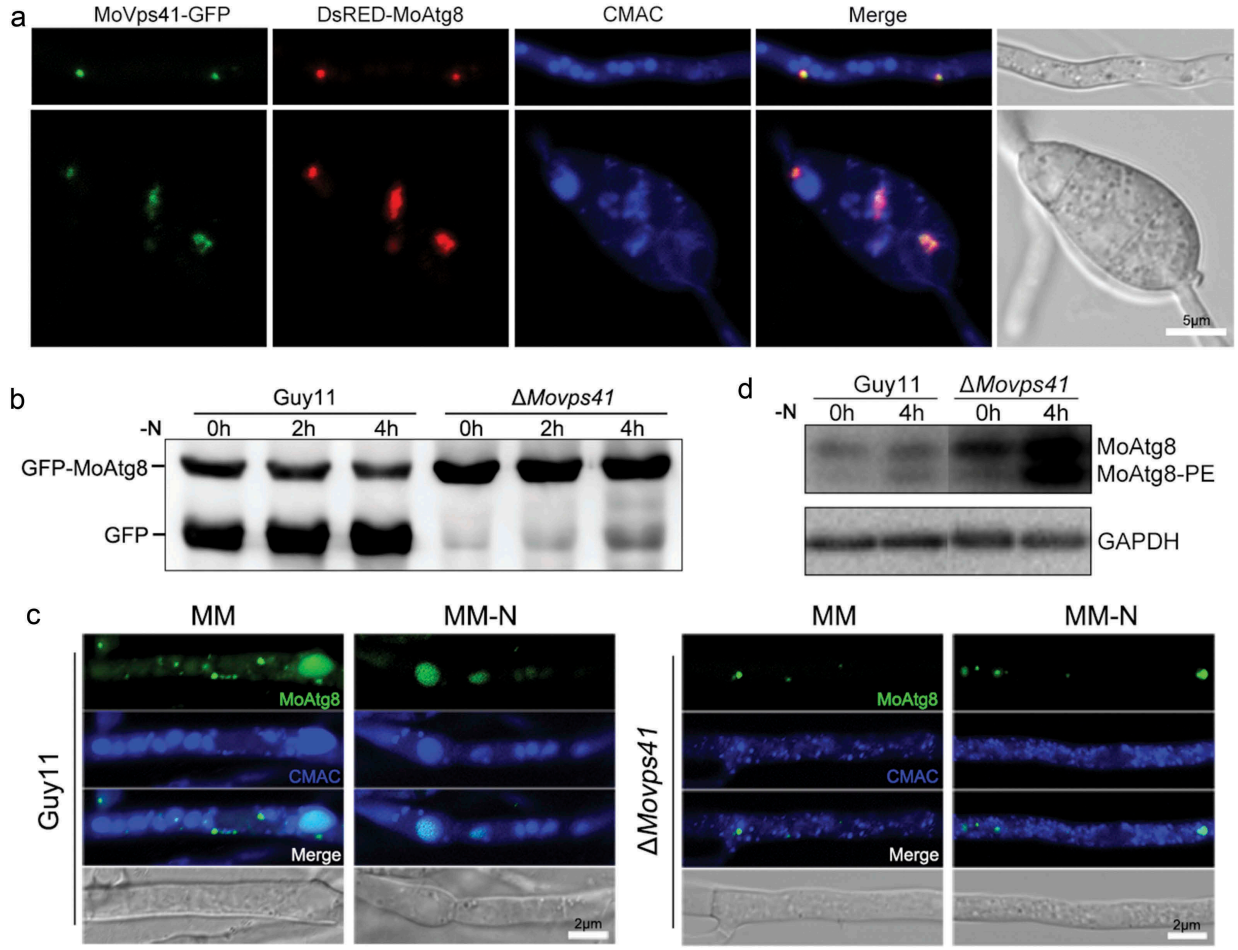
MoVps41 positively regulates autophagic processes. (a). Colocalization of MoVps41 and MoAtg8. GFP-labeled MoVps41 and DsRed-labeled MoAtg8 were observed at the hyphal and conidial stages. Bar = 5 µm. (b). Protein degradation assay of GFP-MoAtg8 by western blotting in Guy11 and Δ*Movps41*. Total proteins of Guy11 and *ΔMovps41* expressing the GFP-MoAtg8 fusion protein were extracted from mycelia at the indicated time after nitrogen starvation and were detected with anti-GFP antibody. (c). Microscopic observation of GFP-MoAtg8 localization. After nitrogen starvation treatment, GFP-MoAtg8 entered the vacuoles and colocalized with the CMAC-labeled vacuole in Guy11. However, GFP-MoAtg8 in Δ*Movps41* still displayed dotted fluorescence outside the vacuoles. Bar = 2 µm. (d). Lipidation of MoAtg8 in Guy11 and Δ*Movps41*. Lipidation of MoAtg8 was induced with nitrogen starvation for 4 hours and detected via western blotting.

In *S. cerevisiae*, Yck3 phosphorylates Vps41 and inhibits its functions in facilitating membrane fusion [56]. Thus, we deduced that the enhancement of autophagy in Δ*Moyck1* might be caused by enhancing MoVps41-mediated membrane fusion between autophagosomes and vacuoles. Although MoVps41 has been shown to be required for vacuole membrane fusion, its roles in autophagy remain elusive. First, to determine whether MoVps41 is involved in the autophagic process, colocalization of DsRed-labeled MoAtg8 and GFP-labeled MoVps41 was monitored. A partial overlap was observed between MoVps41-GFP and DsRed-MoAtg8, implicating the involvement of MoVps41 in autophagy (Figure 7(a)).

Then, a proteolysis assay of the GFP-MoAtg8 fusion protein was monitored to determine whether MoVps41 functioned in autophagic body degradation. When the CM-precultured mycelia of Guy11 were transferred to nitrogen starvation conditions, the levels of GFP-MoAtg8 fusion protein gradually decreased, with an increase in free GFP. In contrast, only a slight degradation of GFP-MoAtg8 occurred in Δ*Movps41* upon nitrogen starvation for 4 hours (Figure 7(b)), indicating that MoVps41 positively regulated autophagy.

Additionally, to determine which process during autophagy induction was impaired in Δ*Movps41*, we observed the transport of the GFP-MoAtg8 fusion protein to the vacuole labeled by CMAC staining. Mycelia of Guy11 and Δ*Movps41* expressing GFP-MoAtg8 were first subjected to incubation in minimal medium (MM). Punctate green fluorescence signals were observed close to CMAC-stained vacuoles in both Guy11 and Δ*Movps41*. After shifting to nitrogen starvation conditions (MM-N) for 4 hours, the green fluorescent dots disappeared, and the GFP signal overlapped with the vacuole in the wild-type strain, indicating normal autophagy-mediated degradation in the vacuole. In contrast, the green fluorescence signal in Δ*Movps41* was still present as punctate forms and stayed outside the fragmented vacuoles (Figure 7(c)). It was further suggested that MoVps41 is essential for the fusion of autophagosomes with vacuoles.

The lipidation of MoAtg8 was also observed in Guy11 and Δ*Movps41*. The results showed that the lipidation of MoAtg8 in Δ*Movps41* was normal as that in wild type, but the amount of MoAtg8 and MoAtg8-PE accumulated in Δ*Movps41*, in contrast to that in the wild type (Figure 7(d)). Taken together, phenotypes related to autophagy in Δ*Movps41* were opposite of those in Δ*Moyck1*, suggesting that MoYck1 might negatively regulate autophagy by controlling MoVps41-mediated membrane fusion between autophagosomes and vacuoles in *M. oryzae*.

### Moyck1 is involved in responses to hyperosmotic stresses

**Figure 8.**
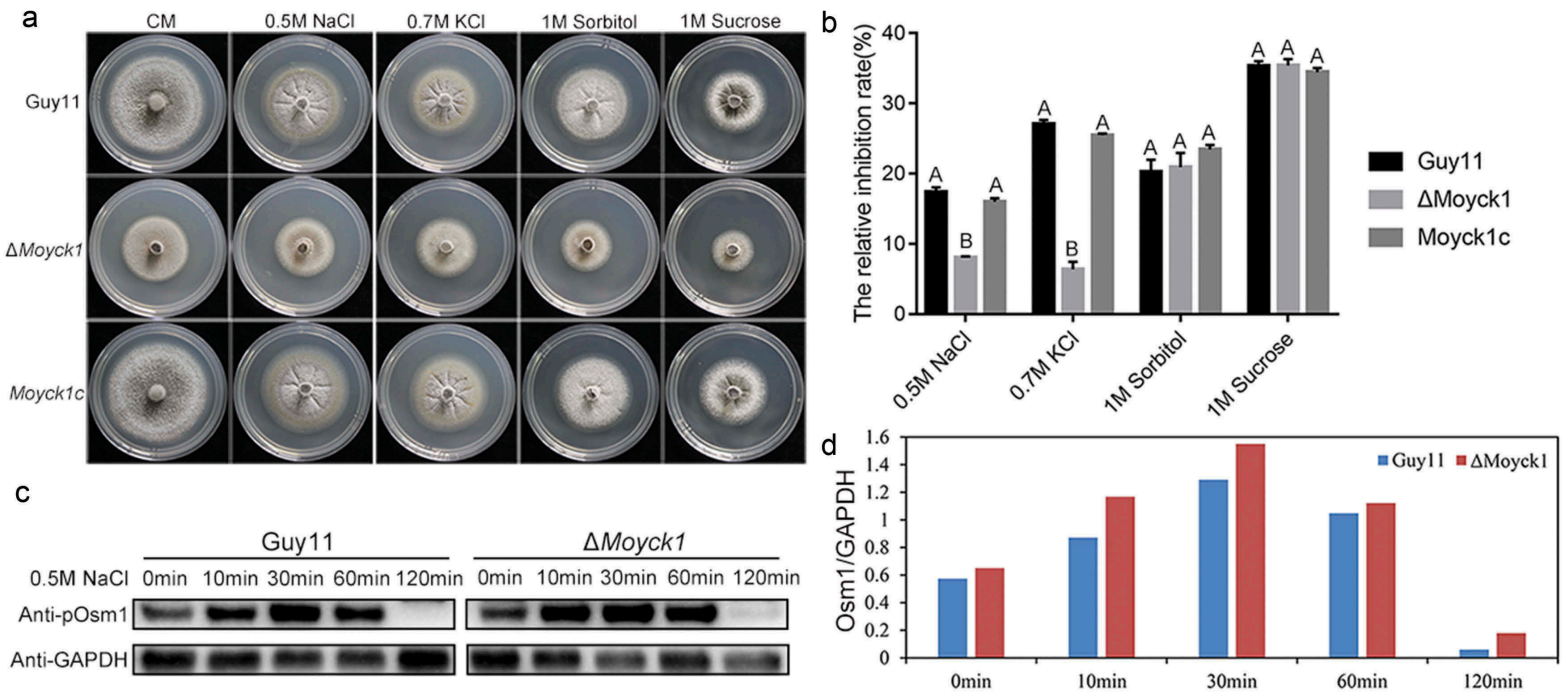
MoYck1 is involved in the osmotic stresses response via regulating the phosphorylation of Osm1. (a). Cultures of Guy11, Δ*Moyck1* and the complemented strain *Moyck1c* on media with hyperosmotic reagents including 0.5 M NaCl, 0.7 M KCl, 1 M sorbitol and 1 M sucrose. Pictures were taken at 7 dpi. (b). The relative inhibition rates of Guy11, Δ*Moyck1* and the complemented strain *Moyck1c* on media with hyperosmotic reagents. Each strain was cultured for three replicates. The columns indicate average values, and the error bars indicate standard deviation. The same letters indicate no apparent differences (Duncan’s test, P < 0.01). (c). Phosphorylation of Osm1. The phosphorylation of Osm1 in Guy11 and Δ*Moyck1* was detected following induction with 0.5 M NaCl at 0, 10, 30, 60, and 120 min. The protein GAPDH was used as a loading control. (d). The relative content of phosphorylated Osm1. The amounts of phosphorylated Osm1 in Guy11 and Δ*Moyck1* were compared with those of GAPDH at the indicated time points.

In *C. neoformans*, Cck1 is required for the hyperosmotic stress response by regulating the phosphorylation level of the MAP kinase Hog1 [15]. To examine whether MoYck1 also functions in hyperosmotic stress response, Guy11, Δ*Moyck1* and *Moyck1c* were tested on CM plates with ionic hypertonic stresses, including 0.5 M NaCl and 0.7 M KCl, and nonionic hypertonic stresses, including 1 M sorbitol and 1 M sucrose. Figure 8(a and b) show that Δ*Moyck1* exhibited greater tolerance to 0.5 M NaCl and 0.7 M KCl than Guy11 and the complemented strain *Moyck1c*, with no significant differences to 1 M sorbitol and 1 M sucrose among strains. These data indicated that MoYck1 plays important roles in adapting to hypertonic stresses.

In *M. oryzae*, the hyperosmotic stress response is regulated by an MAPK Osm1-mediated signaling pathway [4]. We considered that MoYck1 might be involved in the hyperosmotic response via regulation of this pathway. To confirm this, phosphorylation levels of Osm1 in Guy11 and Δ*Moyck1* after treatment with 0.5 M NaCl were monitored by western blotting. As shown in Figure 8(c and d), upon treatment with 0.5 M NaCl, the phosphorylation level of Osm1 in both strains increased before 30 min and then decreased. In contrast, the phosphorylation levels of Osm1 in Δ*Moyck1* were higher than those generated in Guy11 before 120 min, indicating that MoYck1 is required for controlling the phosphorylation of the Osm1 kinase in response to hyperosmotic stress.

### *Moyck1* and *MoVps41* play shared and distinct roles in response to various metal ions

**Figure 9.**
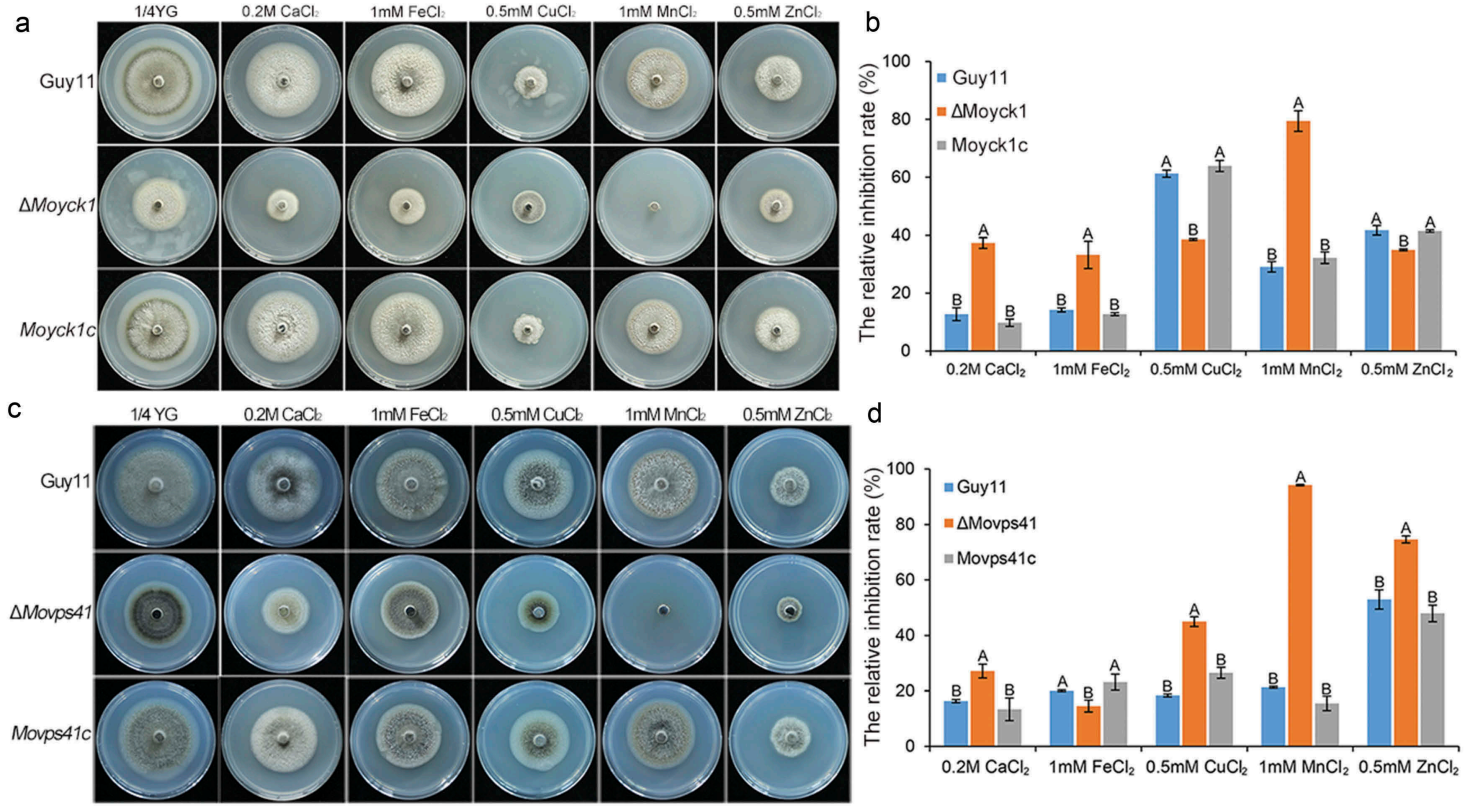
MoYck1 and MoVps41 play crucial roles in the detoxification of heavy metal ions. (a). Mycelial plugs of Guy11, Δ*Moyck1* and the complemented strain *Moyck1c* were inoculated onto 1/4 YG plates with different metal ions. Images were obtained at 6 dpi. (b). The growth inhibition rates were calculated in three independent assays with three replicates each time. Columns labeled with different letters indicate a significant difference determined by Duncan’s test (P < 0.01). (c). Mycelial plugs of Guy11, Δ*Movps41* and the complemented strain *Movps41c* were inoculated onto 1/4 YG plates with different metal ions. Images were obtained at 6 dpi. (d). The growth inhibition rates of Guy11, Δ*Movps41* and the complemented strain *Movps41c* were assessed in three independent assays with three replicates. Columns labeled with different letters indicate a significant difference determined by Duncan’s test (P < 0.01).

Fungal vacuoles play vital roles in maintaining intracellular calcium homeostasis and detoxifying heavy metal ions by sequestering them [29]. Previous studies reported that both MoYpt7 and MoVps41 contribute to protecting cells from damage caused by high-density heavy metal cations. To determine the roles of MoYck1 in response to metal cations, Guy11, Δ*Moyck1* and *Moyck1c* were cultured on 1/4 YG plates supplemented with 0.2 M Ca^2+^, 1 mM Fe^2+^, 0.5 mM Cu^2+^, 1 mM Mn^2+^, and 0.5 mM Zn^2+^. Figure 9(a and b) show that growth of the wild-type strain Guy11 was inhibited at each indicated density of various metal cations, suggesting that the chosen densities of metal ions in our study were beyond physiological conditions. Interestingly, Δ*Moyck1* displayed sensitivity compared with the growth of Guy11 and *Moyck1c* under the same culture conditions. Specifically, Δ*Moyck1* was more sensitive to Ca^2+^, Fe^2+^, and Mn^2+^ but less sensitive to Cu^2+^ and Zn^2+^. In addition, we characterized the metal stress sensitivity of Δ*Movps41*. Figure 9(c and d) show that Δ*Movps41* is more sensitive to Ca^2+^, Cu^2+^, Mn^2+^ and Zn^2+^ but less sensitive to and Fe^2+^, in contrast to the wild type strain and complemented strain *Movps41c*. Our results indicated that the roles of MoYck1 and MoVps41 in response to Ca^2+^ and Mn^2+^ are consistent but converse in response to Fe^2+^, Cu^2+^, and Zn^2+^.

## Discussion

Casein kinases are involved in multiple biological processes via regulating the phosphorylation of various substrates. Deletion of *MoYCK1* causes defects in growth. Growth defects are also reported in the *F. graminearum FgYCK3* deletion mutant [37]. In yeast, Yck1/2 play important roles in glucose sensing and signaling. Under conditions of abundant glucose, Yck1/2 catalyzes the phosphorylation of substrates Std1 and Mth1, which are subject to degradation mediated by the ubiquitin ligase complex SCF^Grr1^ [1]. This would relieve *HXT* gene expression repression and increase glucose uptake [16]. Thus, MoYck1 might also regulate glucose utilization in *M. oryzae*. Alteration of carbon source preference in Δ*Moyck1* partially confirmed this assumption. In addition to growth, we also found that *MoYCK1* is essential for sexual development, which is concordant with studies from homologous genes *FgYck3* in *F. graminearum* and Cck1 in *C. neoformans* [15,37]. In yeast, Ste2, an α-factor receptor localized on the cell membrane, is phosphorylated by Yck1/2 and internalized to transmit mating signaling [58]. Although the Ste2 homolog is not conserved in *M. oryzae*, a similar mechanism might be mediated by other receptors.

Our results show that MoYck1 is involved in infection-related morphogenesis and is essential for full virulence. In *M. oryzae*, virulence is a complicated phenotype involved in the normal development of conidia and appressoria and is not limited to one factor. The *MoYCK1* deletion mutant showed severe defects in conidiogenesis, conidial germination, and appressorium formation. In combination with low appressorium-mediated penetration, all of these defects resulted in reduced virulence of Δ*Moyck1*. Because MoYck1 is involved in regulating many developmental stages, we postulate that MoYck1 might function upstream of developmental processes and is required for the regulation of important cellular processes via the phosphorylation of a great range of substrates.

Autophagy has been confirmed to play crucial roles in the virulence of fungal pathogens. Disruption of autophagy-related core genes impaired autophagy and virulence, indicating the importance of autophagy in fungal infection [8,18]. GFP-MoAtg8 and the lipidation of MoAtg8 are very useful markers to follow autophagy [20]. Interestingly, in our study, we found that lipidation of MoAtg8 and degradation of GFP-MoAtg8 occurred quickly in the Δ*Moyck1* mutant, in contrast to the wild type under nitrogen-lacking conditions. These results indicated that autophagy was enhanced in the *MoYCK1* deletion mutant. MoYck1 is involved in the negative regulation of autophagy. In contrast, disruption of MoVps41 prevented the movement of GFP-MoAtg8 to the vacuolar lumens for degradation and recycling of MoAtg8/MoAtg8-PE when autophagy was induced. The autophagy process was blocked in the Δ*Movps41* mutant. Nevertheless, virulence was impaired substantially in the Δ*Moyck1* and Δ*Movps41* mutants. We reasoned that either excessive or inadequate autophagy contributed to the reduced virulence of *M. oryzae*. Recent reports from other groups support this conclusion. TOR kinase is a conserved negative regulator of autophagy in eukaryotes [59]. Exogenous addition of rapamycin, an inhibitor of TOR kinase, to *M. oryzae* conidial drops activated autophagy but decreased the virulence of the wild type [23]. Deletion of *MoGCN5*, a histone acetyltransferase, also led to autophagy induction and reduced virulence [24]. Collectively, autophagy needs to be tightly regulated during fungal development and infection.

In yeast, Yck3 phosphorylates Vps41 and promotes the dissociation of Vps41 from vacuole conjunctions, which inhibits membrane fusion [56]. In *F. graminearum*, FgYck3 interacts with FgVps41 on FgRab7-expressing endosomes [49]. In combination with previous studies, we presumed that the mechanism by which MoYck1 regulates autophagy may be dependent on MoVps41-mediated membrane fusion between autophagosomes and vacuoles. In our research, we demonstrated that MoVps41 is required for autophagy and has partial colocalization with MoAtg8-expressing autophagosomes. Furthermore, phenotypic changes in autophagy induction are opposite of those in Δ*Movps41* and Δ*Moyck1*, including MoAtg8 lipidation and the observation of autophagy affluxes. However, further experiments to determine the effects of mimicking phosphorylated MoVps41 on autophagy in Δ*Moyck1* need to be carried out to confirm this assumption.

We found that MoYck1 regulates the hyperosmotic response via the Osm1-mediated signaling pathway. These findings are consistent with the functions of Cck1 in *C. neoformans* [15]. Interestingly, it seems that MoYck1 only responds to ionic hyperosmotic stresses, not nonionic hyperosmotic stresses, indicating that these two kinds of hyperosmotic stresses are sensed by different signaling pathways. In *M. oryzae*, the hyperosmotic stress response is mediated by the MAPK Osm1 signaling pathway [4]. In contrast to the wild type, a relatively higher phosphorylation level of Osm1 in Δ*Moyck1* was found, which explains the observation that Δ*Moyck1* is more tolerant to 0.5 M NaCl than the wild type. Because autophagy is activated in Δ*Moyck1*, it is also possible that autophagy acts as a downstream pathway responding to hyperosmotic stress.

The vacuole is also the key organelle for intracellular ionic balance and is responsible for the detoxification of cytoplasmic metal ions [60,61]. A recent study reported that MoVps41 is required for the response to heavy metal ions [35]. Our results are almost consistent with their observations, except for Fe^2+^. This difference may be due to the different densities of metals used. In our studies, we found that Δ*Movps41* and Δ*Moyck1* show different responses to various heavy metal ions, indicating that different detoxification mechanisms are present in fungi. In metal toxicity assays of *S. cerevisiae*, the lowest observable effect level of Mn^2+^ on growth is 1 mM [62]. In our results, the wild-type strain exhibited an approximately 30% growth inhibition rate in response to 1 mM Mn^2+^, showing more sensitivity than yeast. Similarly, neither Δ*Movps41* nor Δ*Moyck1* grew well on 1 mM Mn^2+^, indicating a collaborative role in the detoxification of Mn^2+^. The Δ*Movps41* mutant showed inverse sensitivity to Fe^2+^, Cu^2+^, and Zn^2+^ compared with Δ*Moyck1*, indicating that MoYck1- and MoVps41-mediated vacuolar morphology may be related to the response to Fe^2+^, Cu^2+^, and Zn^2+^. In yeast, Mn^2+^ and Zn^2+^ could be chelated by glutathione and metallothionein, respectively, and then sequestered into vacuoles for detoxification [62]. The sequestration process may be regulated by MoYck1 and MoVps41.

Taken together, our results indicate MoYck1 is involved in growth, asexual or sexual development and virulence in *M. oryzae*. In addition, MoYck1 regulates autophagy, hyperosmotic stress responses and detoxification of heavy metal ions, possibly via regulation of MoVps41, a HOPS subunit. Our study highlights the pivotal roles of casein kinase MoYck1 in phytopathogenic fungi and provides a new potential target for the control of rice blast disease.

## Supplementary Material

Supplemental Material
