# Supplementary material for: The casein kinase MoYck1 regulates development, autophagy, and virulence in the rice blast fungus
Source: Virulence. 2019 Aug 8;10(1):719–33. doi: 10.1080/21505594.2019.1649588 (PMC8647852; doi:10.1080/21505594.2019.1649588)
Supplement: Supplemental Material [file KVIR_A_1649588_SM5907.zip › Supporting information legends.docx]

**Supporting information legends**

**Fig. S1. Schematic construction of *MoYCK1* and *MoVPS41* knockout vectors and Southern blot assay.**

1. Knockout of *MoYCK1* via homologous recombination. **B**. PCR verification of putative null mutants. Both the gene inner fragment and recombination fragment were tested. **C**. Southern blotting of the wild type and Δ*Moyck1.* Genomic DNA of Guy11 and Δ*Moyck1* were digested with *BamHI* and separated on 0.7% (w/v) agarose gels. A single band shifted from 5.9 kb in the wild type to 9.4 kb in Δ*Moyck1* using the probe indicated in Fig. S1A. **D.** Targeted gene deletion of *MoVPS41* by homologous recombination. **E.** Southern blot analysis of *MoVPS41* deletion transformants. The same method was used with the probe displayed in Fig. S1D.

**Fig. S2. qPCR analysis of *GFP-MoATG8* transcription**. Total RNA from samples was extracted with RNAiso plus and reverse-transcribed to cDNA. Expression of *GFP-MoATG8* was detected using a pair of GFP probe primers.

**Fig. S3. qPCR analysis of *MoATG8*.** Transcription of *MoATG8* in Guy11 and Δ*Moyck1* was detected via RT-qPCR.

**Table S1. Primers used in this study.**
