# Supplementary material for: The casein kinase MoYck1 regulates development, autophagy, and virulence in the rice blast fungus
Source: Virulence. 2019 Aug 8;10(1):719–33. doi: 10.1080/21505594.2019.1649588 (PMC8647852; doi:10.1080/21505594.2019.1649588)
Supplement: Supplemental Material [file KVIR_A_1649588_SM5907.zip › Table S1.docx]

**Table S1. Primers used in this study**

| Name | Sequence (5’ - 3’) |
| --- | --- |
| Primers used for knockout and complement. | |
| BAR-F | AGAAGATGATATTGAAGGAGC |
| BAR-R | CTAAATCTCGGTGACGGGCAG |
| VPS41up-F | CCGGGGATCCTCTAGAGAGACCCGACGCACATACTTT |
| VPS41up-R | TGTTGACCTCCACTAAGGCAGACAGGGATTGTTG |
| VPS41dn-F | GGAATAGAGTAGATGGTTATTTCGGGGCGACCAA |
| VPS41dn-R | GGCCAGTGCCAAGCTTCTCTGCGTTAGCCAACCTGT |
| VPS41pb-F | TCGAGACGGGCACTCGTGAC |
| VPS41pb-R | GAGACCACCAACCCGGTCAC |
| VPS41GFPF | GCCGGATCCATCCCCGGGATGTCCGACGAGGAGACTCC |
| VPS41GFPR | CTTGCTCACCATCCCGGGCATTTCCTTACTCCTATCAC |
| HPHF | TAGTGGAGGTCAACAATGAATG |
| HPHR | CATCTACTCTATTCCTTTGCCC |
| YCK1up-F | CCGGGGATCCTCTAGACAATCTTGTCTCCAGGAACG |
| YCK1up-R | TGTTGACCTCCACTAAGGCAGCTCGGTAATCAG |
| YCK1dn-F | GGAATAGAGTAGATGAATCGGTAACTCAGAGGTGT |
| YCK1dn-R | GGCCAGTGCCAAGCTTGGAGCGGTGACTTGAAAC |
| YCK1probeF | TGAGGCAGAGTTCCGTGAT |
| YCK1probeR | AAATGAAAGCCATACAAGC |
| YCK1koyzF | GCAGGTGGCAATCAAGTT |
| YCK1koyzR | AGGTAGTCGTAGTCGGGAGT |
| YCK1upF | GAGCAGCGACAAGGACAA |
| HPHupR | GGCTGATCTGACCAGTTGCC |
| YCK1cF | AATCACTAGTGAATTCCAAGACGATAATGCGAGG |
| YCK1cR | TTCCCGGGGATGGATCCAGAAGAGGGTGGTTGTGAT |
| GFPRTF | GACAACCACTACCTGAGCAC |
| GFPRTR | CAGGACCATGTGATCGCG |
| MoATG8RTF | TGCGAGAAGGTAGAAAAGTCG |
| MoATG8RTR | TGGACGAAGATGAAGATGGC |
| TubulinRTF | ACAACTTCGTCTTCGGTCAG |
| TubulinRTR | GTGATCTGGAAACCCTGGAG |
