# Supplementary figures and images for: The casein kinase MoYck1 regulates development, autophagy, and virulence in the rice blast fungus
Source: Virulence. 2019 Aug 8;10(1):719–33. doi: 10.1080/21505594.2019.1649588 (PMC8647852; doi:10.1080/21505594.2019.1649588)

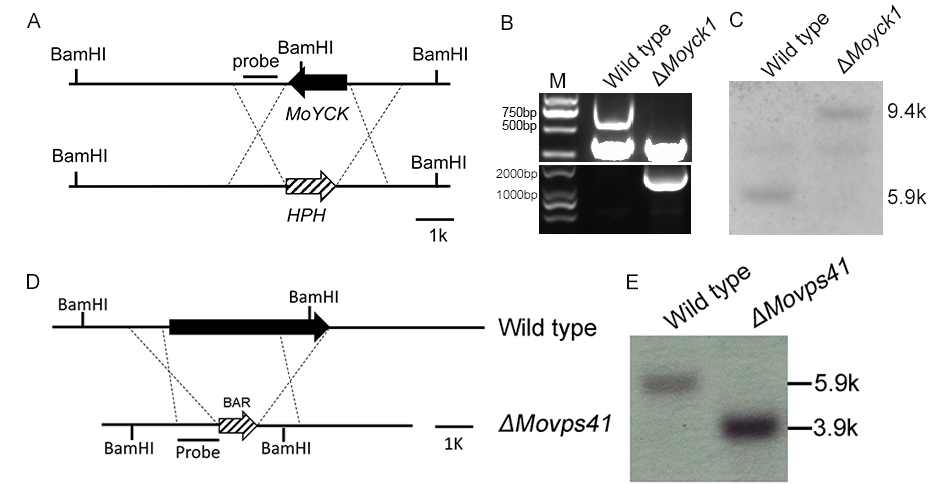

Supplement: Supplemental Material [file KVIR_A_1649588_SM5907.zip › Fig. S1.tif]

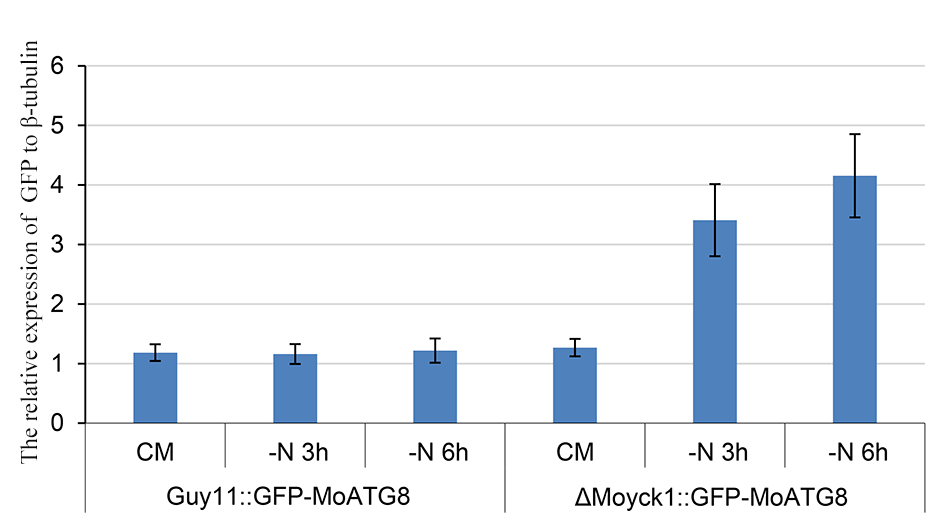

Supplement: Supplemental Material [file KVIR_A_1649588_SM5907.zip › Fig.S2.tif]

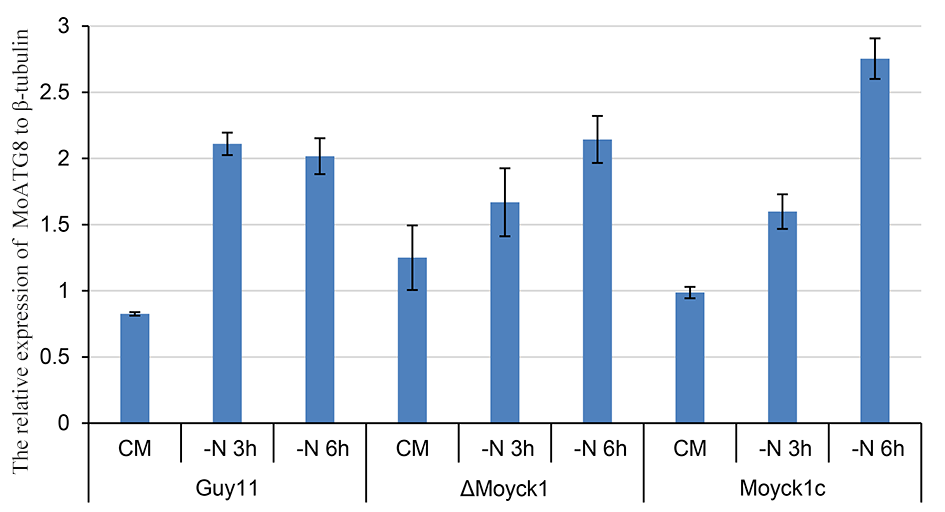

Supplement: Supplemental Material [file KVIR_A_1649588_SM5907.zip › Fig.S3.tif]
